# ECMO use in Germany: An analysis of 29,929 ECMO runs

**DOI:** 10.1371/journal.pone.0260324

**Published:** 2021-12-07

**Authors:** Sven Bercker, David Petroff, Nina Polze, Christian Karagianidis, Thomas Bein, Sven Laudi, Sebastian N. Stehr, Maria Theresa Voelker

**Affiliations:** 1 Department of Anesthesiology and Intensive Care Medicine, University of Leipzig Medical Faculty, Leipzig, Germany; 2 Clinical Trial Centre, University of Leipzig, Leipzig, Germany; 3 Department of Pneumology and Critical Care Medicine, Cologne-Merheim Hospital, ARDS and ECMO Centre, Kliniken der Stadt Köln gGmbH, Witten/Herdecke University Hospital, Cologne, Germany; 4 Department of Anesthesiology and Critical Care Medicine, Faculty of Medicine, University of Regensburg, Regensburg, Germany; IRCCS Policlinico S.Donato, ITALY

## Abstract

**Background:**

Extracorporeal Membrane Oxygenation (ECMO) use is increasing despite limited evidence. The aim of this study was to demonstrate heterogeneity of ECMO use and its association with hospital size and annual frequency in Germany.

**Methods:**

This is a database analysis of all ECMO cases in Germany from 2010 to 2016 using the German Diagnosis Related Groups (DRG) coding system for ECMO.

**Results:**

During the study period, 510 hospitals performed 29,929 ECMO runs (12,572 vvECMO, 11,504 vaECMO, 1993 pECLA) with an increase over time. Mortality ranged between 58% and 66% for vaECMO cases and 66% and 53% for vvECMO cases. 304 (61%) hospitals performed only one ECMO per year. 78%% of all ECMO runs were performed in centres with more than 20 cases per year and more than half of all ECMO runs were performed in hospitals with >1.000 beds. Mortality for vv and vaECMO was highest in very small hospitals (< 200 beds; 70%; 74%) and very large hospitals (>1000 beds; 60%; 62%).

**Conclusions:**

Use of ECMO is still increasing and a substantial proportion of hospitals performs very few ECMO runs. Small hospitals had a significantly higher mortality, but dependence on hospital size and ECMO mortality was irregular.

## Background

Since the first successful treatment with extracorporeal membrane oxygenation nearly 50 years ago [1], there has been a remarkable evolution of extracorporeal cardiopulmonary support [2]. Systems became smarter and complications have been reduced markedly through the introduction of heparin-coated cannulas and circuits.

Today’s ECMO indications are derived from the two main functions of ECMO: providing extracorporeal gas exchange and circulatory support. Venoarterial (va) ECMO is used in patients with cardiogenic shock and venovenous (vv) ECMO and/or vaECMO is used in patients with severe gas exchange disorders [3]. Despite its long history of use, there is only limited evidence for its effectiveness in terms of large randomised controlled trials (RCT). For patients with ARDS, four RCTs have been published and none of them could show a substantial advantage through use of ECMO. The Morris trial [4] and the US ECMO trial of Zapol et al. [5] are only of historical importance. The Cesar trial demonstrated that it was advantageous to be treated in a specialized centre with ECMO [6] availability and also in 2018 Combes et al. who randomised nearly 250 patients with ARDS for ECMO or non-ECMO treatment had to draw the conclusion that ECMO did not lower 60-day mortality [7]. Evidence for treating cardiac shock or even cardiac arrest is likewise insufficient. Aside from some ongoing studies, there is no published RCT showing any benefit of ECMO therapy. Consequently, current guidelines recommend using ECMO in ARDS only as rescue therapy [8] or in patients with severe ARDS with very low quality of evidence [9] and recommendations in cardiogenic shock are also rather cautious [10]. In contrast, the frequency of ECMO use appears to have increased substantially in recent years. Karagiannidis et al demonstrated a marked increase in the incidence of ECMO use between 2007 and 2014 in Germany, the most significant effect being with vaECMO [11]. Data from the extracorporeal life support organization (ELSO) registry demonstrated similar effects [12, 13].

Even if ECMO systems have become smaller and (maybe) safer over time, the technique is associated with its own morbidity and potential complications such as severe bleeding or clotting events, haemolysis, lower extremity ischaemia, compartment syndrome, neurologic complications, acute kidney injury and significant infections, which are severe and frequent [2, 14]. Guidelines for ECMO centres address these demands and recommend ECMO only in tertiary care hospitals [15] with a minimum of 20 ECMO runs per year [8].

With this analysis of a very large nationwide database, we were aiming at demonstrating heterogeneity of ECMO use in Germany and tested the hypothesis that there is an association between hospital size as well as annual frequency and hospital mortality in ECMO therapy.

## Methods

This is a retrospective observational analysis using a nationwide database provided by the Research Data Centre of the Federal Statistical Office of Germany (RDC of the Federal Statistical Office and Statistical Offices of the Länder, DRG statistics, 2010–2016). Every hospital is required to report a core set of data from every inpatient treated which is paid for by the statutory health insurance. These records include information on hospitals, duration of treatment, and mode of discharge, diagnoses and procedures that were stated for billing purposes. Additionally, certain limited information about the patient is given. The local ethics committee (Ethics Committee at the Medical Faculty, Leipzig University) waived the need for informed consent. The Research Data Centre of the Federal Statistical Office of Germany has an additional intrinsic ethics assessment of the project and the data (Research Data Centre of the Federal Statistical Office of the Länder, 65189 Wiesbaden, Gustav-Stresemann-Ring 11, forschungsdatenzentrum@destatis.de). Ethical approval was given here too without informed consent. The mortality given in the data is the hospital mortality of the hospital that performed the ECMO-run.

The German Diagnose Related Groups (gDRG) system allows ECMO therapy to be reported via 4 different billing codes and a variety of sub-codes:

8–852.0 veno-venous ECMO without cardiac assist (vvECMO)
8–852.1 pre-ECMO therapy
8–852.2 pumpless extracorporeal lung assists (pECLA)
8–852.3 veno-arterial and veno-venous-arterial ECMO with cardiac assist (vaECMO)

We postulated that coding for ECMO represents ECMO application with only negligible deviations and therefore analysed the frequency of the different codes. Code 8–852.1 “pre-ECMO therapy” is usually used for patients that ultimately do not receive ECMO but an ECMO machine and a team are on standby. To test the association between mortality and the annual volume of ECMO runs, hospitals were clustered in categories with 1, 2–5, 6–10, 11–20, 20–50 and more than 50 ECMO runs / year. To analyse effects of hospital volume we clustered hospitals in 5 groups: < 200 beds, 201–500 beds, 501–750 beds, 751–1000 beds and >1000 beds.

### Statistical analysis

The Research Data Centre of the Federal Statistical Office of Germany allows researchers to submit statistical scripts, which are then applied to the database, and output is returned. If the output contains details that could compromise data protection law, these parts are blacked-out. The script was prepared using R. The output contained contingency tables, which were then analysed with chi-squared tests and post-hoc comparisons adjusted with Holm-Bonferroni correction. A Cochran-Armitage trend test was originally foreseen for analyses of contingency tables containing ordinal variables, but considered inappropriate due to the unforeseen non-monotone behaviour and thus precluding estimates for risk according to age category. A logistic regression model for mortality was performed with annual volume and hospital size as ordinal variables and adjusting for ECMO type, age and sex. Confidence intervals for proportions were found using a Wilson score.

## Results

**Fig 1:**
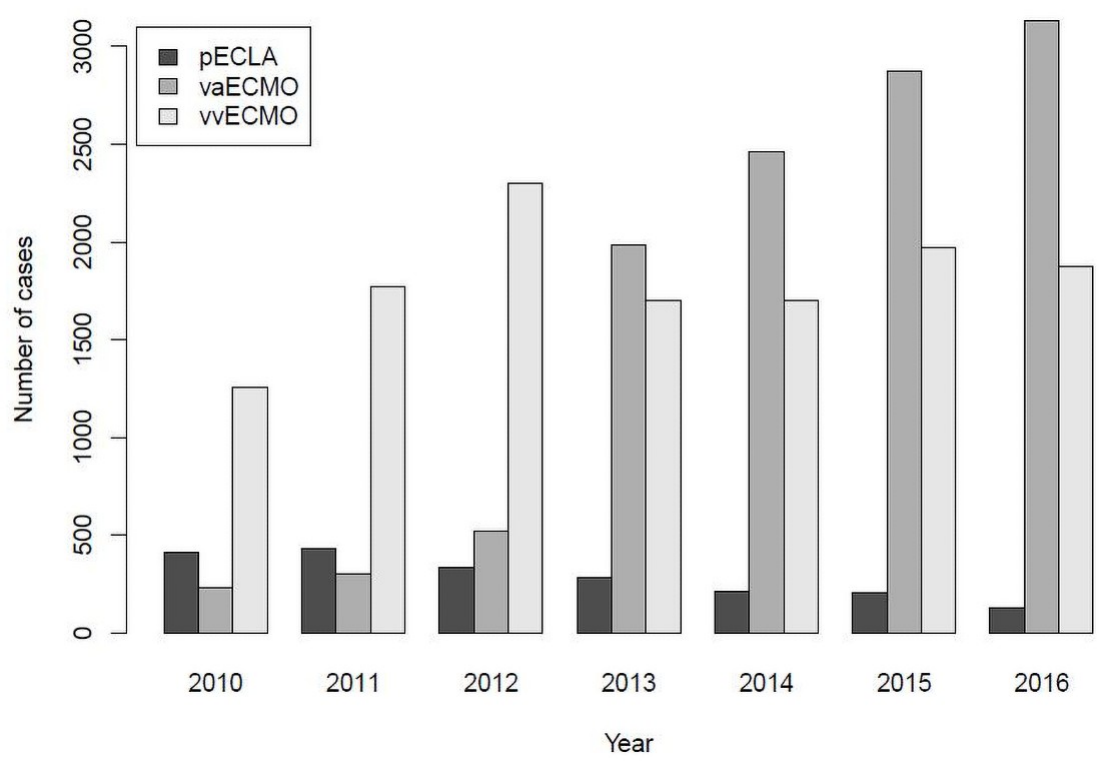
Annual volume of ECMO. Annual volume of ECMO coding in Germany / year.

Between 2010 and 2016, a total of 131,705,648 inpatient data sets were analysed for the use of ECMO. ECMO was coded 29,929 times. Among them were 12,572 vvECMO applications (8–852.0), 11,504 vaECMO applications (8–852.3), 1993 pECLA applications (8–852.2) and 3,039 preECMO therapy. For 821 patients both vaECMO and vvECMO were documented. These patients were excluded from further analysis as interpretation of the data would have been very difficult and the overall number of 821 comprise only 2% of the total. We further excluded preECMO therapy as these patients did not actually receive ECMO therapy, which was simply on stand-by. There was a notable increase in ECMO therapy during the study period, primarily due to a more than 10-fold increase of vaECMO (Fig 1).

### Heterogeneity of ECMO application

There was considerable heterogeneity of ECMO application concerning regional and inter-hospital differences.

During the study period 510 German hospitals performed ECMO. 304 of them had only one case per year. A total of 457 hospital performed only 20 or fewer ECMO runs per year (Table 1). Only 53 hospitals reported more than 20 ECMO runs per year. However, 10% of the hospitals performed 78% of all ECMO runs.

**Table 1:** Number of hospitals performing ECMO arranged in categories (mean annual ECMO volume over all 6 years) and total numbers of vvECMO and vaECMO per hospital category from 2010–2016.

	1	2–5	6–10	11–20	21–50	>50
Hospitals performing ECMO [n]; % of all hospitals	304 (60%)	92 (18%)	33 (6%)	28 (5%)	26 (5%)	27 (5%)
Number of VA ECMOs [n]; % of all ECMO runs	139 (1%)	359 (3%)	471 (4%)	1272 (11%)	2398 (21%)	6865 (60%)
Number of VV ECMOs [n]; % of all ECMO runs	327 (3%)	740 (6%)	761 (6%)	1272 (10%)	2694 (21%)	6778 (54%)

For example, 304 of the hospitals averaged 1 or fewer ECMO runs per year (column 1) and these hospitals performed 139 VA and 327 VV ECMOs over the entire study period of 6 years.

More than half of all ECMO runs was performed in hospitals with >1.000 beds (Table 2). There was a smaller peak for all ECMO types at the hospitals with 201 to 500 beds, presumably representing “stand-alone” heart centres.

**Table 2:** Total ECMO treatments and mortality by hospital size from 2010 to2016.

	Number of beds				
	1–200	201–500	501–750	751–1000	>1000
**Total ECMO treatments** (in % of all)					
pECLA	4%*	18%	12%	9%	57%
vaECMO	12%*	20%	7%	11%	51%
vvECMO	7%*	15%	8%	11%	59%
**Mortality** [n]; (%)					
vaECMO	994 (74%)	1356 (60%)	512 (64%)	696 (55%)	3983 (68%)
vvECMO	660 (70%)	1031 (56%)	545 (52%)	684 (50%)	4566 (62%)

Hospital size by number of beds in categories.

* p-values < 0.001 compared to any of the other categories.

### Mortality

Mortality ranged from 58% to 66% in vaECMO without a trend over time (Table 3). Between contiguous years, only the change from 58% in 2012 to 65% in 2013 was significant, with an estimated difference of 6.6 percentage points (95% CI 1.8 to 11.5, p_adjusted_ = 0.036). Mortality decreased from 66% to 53% in vvECMO patients between 2010 and 2016 with the change of −5.7 percentage points from 2012 to 2013 being the only significant change (95% CI −2.6 to −8.9, p_adjusted_ = 0.0017). Mean mortality in all patients was 64% (va) and 60% (vv).

**Table 3:** Mortality (95% CI) for vvECMO and vaECMO.

	2010	2011	2012	2013	2014	2015	2016	total
vaECMO	63%	63%	58%	65%	66%	67%	66%	65.6%
	(57–69)	(57–68)	(54–62)	(62–67)	(64–68)	(65–69)	(64–68)	(65–66)
vvECMO	66%	65%	63%	57%	58%	56%	53%	59.6%
	(63–68)	(63–68)	(61–65)	(55–60)	(55–60)	(54–58)	(51–55)	(59–60)

Very small hospitals with up to 200 beds had the highest mortality rate (74% vaECMO and 70% in vvECMO, Table 2) followed by hospitals with more than 1000 beds. The proportion of deaths in hospitals with 1–200 bed is significantly higher than in any of the other categories both for va and vvECMO (all p-values < 0.001).

**Fig 2:**
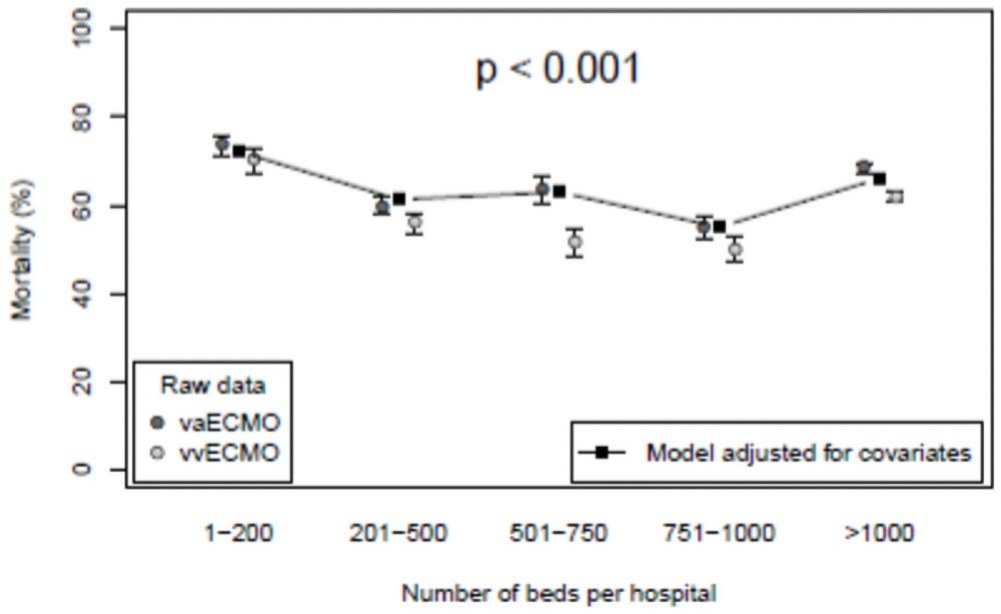
Mortality and hospital size. Mortality for patients is shown according to ECMO hospital size. The points connected by lines are taken from a logistic regression model adjusting for ECMO type, age and sex. A 61–70 year old, male vaECMO patient is depicted, but this choice does not affect the relative positions.

Hospitals with an average of ≥ 6 ECMO cases per year had a mean mortality of around 60% in vvECMO and between 60 and 70% in vaECMO. For both techniques hospitals with lower ECMO frequency had a significantly lower mortality. However, in the logistic regression model we found both annual ECMO volume and hospital size were independently associated with mortality (p < 0.001 for both) after adjusting for ECMO type, age and sex. ECMO volume is a six category ordinal variable meaning that a fifth order polynomial was fit in the regression model. The linear and quadratic terms were significantly positive and negative, respectively (p < 0.001 for both, see Fig 2). Hospital size is an ordinal variable with five categories meaning a fourth order polynomial was fit in the regression model. All four coefficients were significant (all p < 0.001), resulting in erratic behaviour from one category to the next (Fig 2).

### ECMO and age category

There has been a noticeable shift toward treating older patients since 2010 (Table 4). In particular, there was a jump between 2012 and 2013 in the number of patients over the age of 80, treated with ECMO.

**Table 4:** Number of ECMO cases and age category.

Age category	Year						
	2010	2011	2012	2013	2014	2015	2016
18–30	165 (10%)	182 (8%)	225 (7%)	264 (6%)	253 (5%)	271 (4%)	289 (4%)
31–60	982 (24%)	1239 (23%)	1511 (21%)	2129 (21%)	2165 (18%)	2484 (18%)	2572 (17%)
61–70	435 (33%)	596 (34%)	776 (33%)	958 (29%)	1232 (30%)	1478 (31%)	1558 (31%)
71–80	346 (26%)	473 (26%)	661 (28%)	934 (28%)	1195 (29%)	1382 (29%)	1405 (28%)
> 80	48 (7%)	86 (9%)	112 (9%)	262 (16%)	385 (19%)	415 (18%)	460 (19%)
% >70	20%	22%	24%	26%	30%	30%	30%
% > 80	2%	3%	3%	6%	7%	7%	7%

The numbers in brackets indicate the percentage of ECMO cases by life-year, e.g. the 165 cases in 2010 for 18–30 year old are divided by 13. The category “>80” is treated as making up five life-years.

## Discussion

With this study, we demonstrated:

- ECMO use in Germany is still increasing. This is mainly due to the massive increase in the use of vaECMO. ECMO therapy in very old patients increased between 2010 and 2016.
- Very small hospitals had a significantly higher mortality than hospitals with more than 200 beds, but dependence on size was irregular
- Mortality after ECMO therapy increases with annual frequency.

The increase in ECMO applications in Germany has already been described by Karagiannidis et al. [11] We demonstrate a further increase in vaECMO and overall stable use of vvECMO since 2011 with a single peak in 2012 (Fig 1).

This study is the first to characterize hospitals performing ECMO in Germany. Overall, 510 German hospitals performed ECMO between 2010 and 2016, and 304 of them had only one case per year on average. However, 89% of all ECMO treatments were performed in hospitals with an annual volume of more than 10and 78% in hospitals with more than 20 annual runs.

We observed a hospital mortality of 64% in vaECMO and 60% in vvECMO. Between 2010 and 2016, vaECMO mortality remained on a high level whereas there was a trend towards a lower mortality in vvECMO. This real-world ECMO mortality is higher than values published by specialised centres during controlled studies in the last 25 years. For vvECMO, centres worldwide reported mortalities between 34% and 50% [16–19] which was even lower during the H1N1 pandemic in winter 2009 / 2010 [20, 21].

There are a few potential reasons why this real-life mortality in our study is higher than that published so far: (a) studies use defined inclusion criteria that often exclude patients with poor prognosis; (b) ECMO centres which report to the ELSO registry are not representative of all hospitals using ECMO. In 2019 20 German Centres were ELSO members whereas 510 hospitals ran at least 1 ECMO in 2016; (c) Individual outcome studies are usually performed in centres with high expertise and a presumed better outcome.

Mortality of vvECMO treated patients suggests a decrease over the last years. As ECMO mortality depends on a broad variety of effects we can only hypothesize if this might be a result of growing experience, growing safety of devices or the selection of patients which of course might also be a function of experience. However, whilst mortality in vvECMO decreased, mortality in vaECMO did not. The increase of the proportion of aged (>80 years) patients from 2010 (2%) to 2016 (7%) might be an indicator for a trend towards the treatment of more high-risk patients and therefore, might explain the increase of mortality in vaECMO.

We could not confirm a clear volume-outcome relationship in ECMO therapy. According to current recommendations [8]. ECMO centres should perform at least 20 ECMO cases per year. This was inferred from a retrospective ELSO database analysis [12]. However, there is broad evidence from other procedures that a high hospital volume of cases is associated with lower mortality. This has been shown for a variety of procedures and medical conditions [22]. We do not doubt the meaningfulness of experience, but based on our study we postulate that strong evidence to recommend a specific minimum number of treatments is lacking. The increased mortality in high-volume centres and large hospitals might easily be explained by a larger volume of complex patients and a higher proportion of patients who were already referred from other hospitals [16, 23, 24].

One of the main limitations of our analysis is the lack of risk adjustment and therefore, it is likely that hospitals with smaller volumes included patients with a higher probability of survival or patients were transferred to larger hospitals (accounting for “survival” if they were transferred alive on ECMO) where they might later have died. The database used from cases reported to the Research Data Centre of the Federal Statistical Office of Germany does not allow for a cross references. The same patients in two hospitals count as two separate cases.

In conclusion, we could demonstrate that

1. Real-life mortality of all ECMO runs conducted in Germany is higher than that of selected groups published in studies.
2. In Germany a broad variety of hospitals of all sizes perform ECMO and a substantial part performs very few.
3. Small hospitals had a significantly higher mortality, but a clear correlation between hospital size and ECMO mortality in this unadjusted database could not be shown.
